# Exploring the role of translators’ emotion regulation and critical thinking ability in translation performance

**DOI:** 10.3389/fpsyg.2022.1037829

**Published:** 2022-11-17

**Authors:** Shufang Cheng

**Affiliations:** School of Foreign Languages, Zhengzhou University of Aeronautics, Zhengzhou, China

**Keywords:** critical thinking, emotion regulation, translation performance, psychology, translation studies

## Abstract

In recent years, the field of psychology has received more attention from researchers that work in the area of translation studies. This review set out to delve into the role of translation students’ critical thinking, as a construct of cognitive psychology, and emotion regulation, as a positive psychological construct, in translation performance. The positive and significant relationship between translation students’ critical thinking skill and their translation performance has been verified in the literature. Moreover, studies have revealed that emotion regulation and its regulator components, such as emotional intelligence, intuition, resilience, and professional expertise can significantly influence translation performance. This review can be beneficial for translation trainees, translation trainers, and curriculum designers to raise their awareness about the role of critical thinking and emotion regulation in translation studies.

## Introduction

Psychology is a very broad subject that mainly deals with human behavior, thoughts, reasoning, and perceptions. Psychology can be used in numerous issues that constitute daily life, including the examination of internal mental processes and the improvement of higher-order thinking skills to effectively cope with different aspects of life such as education. Therefore, psychology plays a significant role in our lives, regardless of our knowledge about it (Al-Jarf, 2022). It has been ordered into a few sub-branches, including clinical, cognitive, developmental, evolutionary, forensic, health, occupational, social, and neuropsychology. Centrally involved (among these branches of psychology) in translation studies is cognitive psychology. Due to the emphasis on internal mental processes, the cognitive approach caused a revolution in the science of psychology and became the dominant approach in psychology in the late 1970s (Wang, 2020). According to McLeod (2007) cognition refers to the knowledge. He asserted that psychologists examine cognition which is “the mental act or process by which knowledge is acquired” (p. 73). This branch of psychology inspects internal psychological developments, including the way of thinking, perceiving, communicating, remembering, and learning. Investigating the role of cognition on internal mental processes and higher order thinking skills like critical thinking skills, the important effect of these skills on all aspects of our communication from listening to writing, and the important impact of these skills on the process of translation and the quality of the final product of this process have drawn the attention of investigators.

Critical thinking is considered a major and important construct of cognitive psychology. This primary area of cognitive psychology, despite its steadily increasing importance over time and its area of study, that is, the study of internal mental processes reflects a relatively new, stimulating, and very attractive research perspective (Wang, 2020). As Sternberg (1986) stated, critical thinking is “the mental processes, strategies and representations people use to solve problems, make decisions, and learn new concepts” (p. 3). There have been some investigations about the association between critical thinking and different kinds of cognitive abilities from writing to reading ability (e.g., Yildirim and Soylemez, 2018; Mbato, 2019; Bean and Melzer, 2021; Nejad et al., 2022). Moreover, critical thinking as an important component of cognitive psychology can have an important role in every process that involves cognitive and metacognitive skills such as translation. According to National Network for Translation, a competent translator must have various skills as: professionalism, networking skills, attention to detail, flexibility/adaptability, organizational skills, writing skills, general knowledge, analytical skills, subject knowledge, curiosity, excellent knowledge of the foreign language, IT skills, picking up new ideas quickly, good cultural awareness, love of reading and research skills. Most of these characteristics such as flexibility/adaptability, organizational skills, attention to detail, analytical skills, research skills, and curiosity require deep understanding and thinking skills (Azin and Heidari Tabrizi, 2016). Therefore critical thinking is significant in studying translation performance. This review aims to shed light on the studies of cognitive psychology to scrutinize the correlation between critical thinking skill and translation quality.

Translation students are constantly exposed to different types of texts which require them to be able to use critical thinking skills as well as translation skills simultaneously. Critical thinking skills govern the process from the beginning, from the time the translator starts reading the source text, until the end, which is the production of the end result, the target text. A translator who has the ability to think critically does indeed have the ability to examine her/his given choices and their implications. This translator makes choices pertinently and decides on how to use her/his various competencies. To achieve this, s/he should have the power of higher-order thinking, or in other words, have the ability to think critically. Critical thinking helps a translator to go further than just the surface of the text and to think deeply, have an overview of a text and find the whys and nature of the text. Therefore s/he can easily analyze, interpret, evaluate, and make decisions (Mohseni and Satariyan, 2011).

Translators are constantly dealing with a complex interaction between text, reader, and the first and second languages, and they need to understand the main idea and concept of the text that should be translated. In order to comprehend the main idea of the text and facilitate this complex interaction, they require critical thinking skill which is required for solving problems, making judgments, learning new notions, and controlling one’s feelings (Saud, 2020). Critical thinking skill, as a cognitive skill, is used beyond the mere understanding of the main idea of the source text. Neubert (1997) argues that “in order to achieve a satisfactory target text, the established rules of correspondence between L1 and L2 need to be criticality extended” (p. 20). Kussmaul (1995) believes that “criticality is not a talent given to the select few, but that as basic features of the human mind, anyone can be critical when they transfer source texts to target texts” (p. 52). Due to the important role of these skills in the process of translation and their high degree of contribution to the understanding of the concept of the text as an important step in the process of translation, it is important to delve into the association between critical thinking as an indispensable part of the translation process and the quality of the translated text as the final product of this process.

Another important element in translation is the translators’ emotional states. Translators’ positive emotions have crucial roles in translation. According to Derakhshan et al. (2022), the broaden-and-build theory supported positive psychology, and it justifies that experiencing positive emotional constructs, such as enjoyment, love, happiness, engagement, resilience, grit, self-efficacy, and emotional regulation “broaden people’s momentary thought-action repertoires and build their enduring personal resources” (p. 2). Positive psychology, as a modern approach to learning of foreign language, has been expanded in recent years (e.g., Wang et al., 2021; 2022; Xie and Derakhshan, 2021). Emotion regulation is a related concept to positive psychology (Derakhshan et al., 2021). Gross (2007) also indicated that emotion regulation refers to “shaping which emotions one has, when one has them, and how one experiences or expresses these emotions” (p. 6). Another objective of this review is to investigate translation students’ emotion regulation, the factors that regulate translator emotions, and their influence on translation performance. Studying these psychological features of translators and their effects on translation performance can pave the way for solving potential problems in translation performance and promoting its quality.

## Review of literature

### The concept of translation

The English term translation is taken from Old French, and it occurs between two unlike languages which are called source language (SL) and target language (TL); also, it might be either written or oral translation (Munday, 2016). In a similar vein, Pym (2014) refers to Start text “as the one we translate from, and to the target culture as the translation produced; then translating is set of the process leading from one side to the other” (p. 1). Moreover, according to Newmark (1988) translation is “rendering the meaning of a text into another language in the way that the author intended the text” (p. 5). House (2015) mentioned that translation results from a linguistic-textual procedure, in which the source language is re-contextualized in another language. Erton (2020) stated that translation is the process of conveying messages across cultural and linguistic barriers, and it is remarkably communicative. Additionally, Jakobson (2004) proposed some definitions of translation for three types of translation, including intra-lingual, inter-lingual, and inter-semiotic. He defined intra-lingual translation as translating the verbal signs of the same language by using the other existing signs in that language. He also mentioned that inter-lingual translation is described as the employment of verbal signs in one language by applying the verbal signs of the other language that we are supposed to translate. Moreover, they asserted that inter-semiotic translation is the employment of verbal signs of one language by using non-verbal signs.

Tymoczko (2018) asserted that translation is considered a multifaceted language-related cognitive activity. According to Lefevere (1992), there are factors influencing the performance of translator’s in translation, such as cognitive, metacognitive, emotional, and cultural factors. Accordingly, Alves and Gonçalves (2013) mention that verbalizing for communication will cause some alterations in the cognitive situation; moreover, translation can be explored from several points of view that can be linguistic, discursive, cultural, social, political, and emotional. Lahodynskyi et al. (2019) also underscored the considerable skills for becoming a successful translator. He listed some features of good translators, including higher linguistic proficiency, extensive cultural background, analytical mind, higher proficiency in the subject matter, along with numerous significant potentials, such as outstanding memory, flexibility, and time-management skills. House (2015) stated that translation considerably depends on numerous extra-linguistic features. He mentioned that the relationship between linguistic-textual features and extra-linguistic contextual factors makes translation such a complicated process. The emergence of translation psychology during the 1990s changed the focus of investigators from the translation process to the translators and their individual cognitive and psychological differences (Jääskeläinen, 2012). Therefore, translators’ cognitive and psychological constructs, as the causal factors of complexity in the translation process, have drawn the attention of many investigators (Mahdy et al., 2020).

Emotions have, in general, been indicated as a noteworthy feature in information processing in translation, and negative and positive emotions result in diverse processing styles (Rojo and Ramos, 2018). The emotional states of translators have also affected the creativity and quality of the translated text (Jääskeläinen and Lacruz, 2018). The regulation of emotional states develops the thinking process and attention span by the enhancement of logical thinking and problem-solving skill (López and Naranjo, 2021).

### The concept of emotion regulation

According to Gross and John (2003), emotion regulation refers to processes through which individuals control their feelings, such as resentment or apprehension. They mentioned that emotion regulation highlights the increasing, maintaining, or decreasing positive and negative emotional states. According to Gross (1998), emotion regulation is “the processes by which individuals influence which emotions they have, when they have them, and how they experience and express these emotions” (p. 275). Gross and John (2003) described emotion regulation as a cognitive model dealing with controlling one’s own emotions (i.e., self-emotion regulation) without regard for controlling the feelings of others.

Two main emotion regulation strategies affecting individuals’ behavior are cognitive reappraisal and expressive suppression (Gross, 1998). According to Gross and John (2003) cognitive reappraisal refers to “a form of cognitive change that involves construing a potentially emotion-eliciting situation in a way that changes its emotional impact” (p. 349). Bielak and Mystkowska-Wiertelak (2020) also mentioned that cognitive reappraisal includes the reinterpretation of the meaning of a stimulus that results in the regulating of the emotion. They mentioned that cognitive reappraisal, as a cognitive, modification strategy, is used for decreasing the response to negative emotions. On the other hand, Gross (1998) stated that expressive suppression “is a conscious inhibition of ongoing emotion expressive behavior” (p. 226). Gross (2015) noted that expressive suppression refers to not showing others what one is feeling. He asserted that it is a type of strategy that individuals employ to stop expressing negative emotions.

### The relationship between emotion regulators and translation performance

Numerous translation researchers have recently approved the significance of translators’ emotions for investigating translation development and have begun to inspect the influence of emotion on translators’ performance (e.g., Hubscher-Davidson, 2018; Lehr, 2020; Hunziker Heeb et al., 2021). The statement that decision-making in translation is not simply the outcome of pure rational thought motivates investigators to explore the effect of psychological states on the translation process (Hubscher-Davidson and Lehr, 2021). In addition to the investigation of emotional causes in the translation environment, the latest research has focused on the translator’s ability to regulate emotions when translating (Hoffmann et al., 2020). Cifuentes-Férez and Fenollar-Cortés (2017) investigated the effect of student translators’ emotional management skills on translation performance. They considered three constituents of emotional management skills, including emotion regulation, emotional expressivity, and self-esteem. They underscored the significance of emotion regulation in the quality of learners’ translation performance. Their study implicated that translation students should practice hiding or inhibiting negative emotional states and responses to perform better in translation tasks. Using Gross and John (2003) Emotion Regulation Questionnaire, they showed that translators who consistently suppress their feelings outperform in translation, and the regulation of translators’ emotions regularly affects their quality of performance. Moreover, their study indicated that the interaction of emotion regulation and emotional expressivity can significantly predict translation quality. It means that translators who inhibit expressing their negative emotions and inhibit their emotional states are more likely to have a qualified translation. Hunziker Heeb et al. (2021) found the significant effect of translators’ emotion regulation on decreasing the cognitive load of translation. They argued that translators’ positive and negative emotional states have large amounts of cognitive load during task performance, and regulating translators’ emotions can inhibit them from producing translations with poor quality. Their study contributes to understanding how translators cope with the additional challenges of emotional aspects of their work and provide insights into how competencies such as emotion regulation might be included in the training.

Rojo (2017) highlighted the role of personality traits and degree of professional expertise, as two significant emotional regulators in determining the performance of translation. He asserted that only a few personality traits have been indicated as significant constructs in regulating translators’ emotions during translation performance. He also asserted that intuition, emotional intelligence, and resilience are regarded as the emotion regulators of translators in translation performance (Rojo, 2017). His study also implicated that upgrading translators’ ability to regulate emotions requires the awareness of the consequences of negative and positive emotions on translation performance and of the factors that can mediate the effects. Rojo and Ramos (2016) studied the impact of personality traits, such as resiliency, on translation performance. They repeated Lehr (2013) methodology. They indicated that translators with low levels of resilience, who were accustomed to getting reproach from their customers, are likely to prevail over the consequences of negative feedback more competently than translators with higher levels of resilience. Their study verified evidence from Lehr’s work, pointing to a differential impact of emotions on different facets of translation performance and signifying that various emotions may trigger diverse processing styles. Rojo and Ramos (2018) examined the role of expertise in emotion regulation and its outcomes for translation quality. They compared the performance of translation students and professional translators through Lehr (2013) methodology, and they assessed trait variation in psychological resilience. Their study revealed that personality factors, such as resilience and expertise level, are influential in controlling emotional states and directing translational behavior, and they may enhance translation quality.

In another study, Hubscher-Davidson (2013) found that emotional intelligence, as a type of personality traits, is significant in controlling translators’ behavior and can be effective in the quality of translation. Emotional Intelligence is regarded as one of the most significant factors that seemingly regulates translators’ minds and contributes to them to be creative in translation tasks (Ebrahimi et al., 2016). Hubscher-Davidson (2013) also found a difference between literary and non-literary translators regarding their emotional intelligence scores. Her study revealed that higher emotional intelligent translators are inclined to regulate their emotions and control the affective nature of texts. Their study implicated the significant role of emotional intelligence to gain a deeper understanding of translation and interpreting processes. Having used Waddington (2001) translation assessment rubric, Ghobadi et al. (2021) investigated the predictability power of individual cognitive-emotional differences, including working memory, emotional intelligence, and, tolerance of ambiguity, in translation performance. They found that the interaction of these cognitive-emotional components predicts translation performance. They also mentioned that higher emotional intelligent translators are more aware of linguistic and non-linguistic relations in a language, and they can appropriately render the source language to the target language, particularly in oral translation. Their study implicated that individual cognitive differences could have some potential effects on translation performance. Moreover, translation trainers should pay attention to students’ internal psychological traits when designing translation-training programs so that they could align the programs with the strengths, weaknesses, and personality characteristics of their trainee. Using structural equitation modeling, Ghaemi and Bayati (2022) explored the role of experienced translators’ burnout and emotional intelligence in translation competence. Their findings demonstrated that, in contrast to burnout, emotional intelligence is significantly correlated with translation competence. They argued that emotionally intelligent translators with higher levels of interpersonal, adaptability, and stress management capabilities, are likely to have more competence in translation. Their study has some implications for translator trainers. The positive relationship found between emotional intelligence and translation competence of language learners can encourage policymakers and curriculum designers to equip language teachers with appropriate training programs to foster the emotional competencies of their translators. Some helpful techniques, which can be used to increase emotional intelligence in the classroom, include discussion, listening to light music, watching emotional clips, self-disclosure, designing questionnaires, reading literature, and psychological texts. Also, Ferdowsi and Razmi (2022) examined the relationship between emotional intelligence, self-efficacy, creativity, and simultaneous translation quality. Their study indicated that simultaneous translators’ emotional intelligence significantly correlates with their self-efficacy and creativity during their translation performance.

Hubscher-Davidson (2013) highlighted the role of translators’ intuition as an emotional regulator in predicting students’ and professional translators’ translation performance. Benjamins Vottonen and Kujamäki (2021) investigated the extent of learners’ reliance on their theoretical knowledge of translation in their justifications and use of the meta-language of the field. Using transcribed retrospections, students were asked to express their opinion when translating texts. Their findings indicated that around one-third of all decision-making in translating complex sentences and unknown words is based on their intuition, and the use of meta-language is scarce. They argued that learned principles and theories can make ‘intuition’ among student translators, and it can turn into tacit, implicit knowledge that is demanding to verbalize. This implies that non-conscious and intuitive decision-making are significant features in the student translators’ translation performance. They also mentioned that student translators are inclined to use their intuition facing translation’s predominant, difficult problems.

Professional expertise is also regarded as a decisive component of emotion regulation. Rojo (2017), in analyzing the effect of emotions on translation performance, found that personality traits and translators’ emotions can be regulated by the translator’s professional expertise. Angelone and Shreve (2011) elucidated the role of translators’ expertise in emotional regulation. They mentioned that expert translators employ metacognition to regulate their emotions. They argued that professional translators’ metacognition can inhibit the negative emotions related to ambiguity controlling translation, resulting in high-quality translation. Mellinger (2019) asserted that student translators, with higher metacognitive skills, tend to employ problem awareness and problem-solving behaviors during the translation task. Moreover, they tend to have higher levels of translation expertise. His findings contribute to the understanding of specialized translation pedagogy and illustrate how metacognitive behavior can change as a result of coursework. Whyatt (2018a) also defined a translator as an individual who uses his professional expertise to produce translations with acceptable quality. Whyatt (2018b) also indicated that expertise in translation refers to features, including “fewer external resources, shorter problem-solving pauses, fast text production, and high-quality target texts” (p. 260). His findings showed that the frequency of problem-solving pauses can differentiate professional translators from novice ones in the paraphrasing task. Moreover, his study showed that expertise in translation leads to superior performance in paraphrase and in bilingual knowledge management in the context of translation as a cross-language task. The dual nature of translation expertise can be optimized in translation training programs and in individual self-development by deliberate practice.

### Critical thinking and features of critical thinkers

Many investigators have provided numerous definitions for critical thinking. Yulian (2021) regarded critical thinking skills as the main cognitive process dimension in Bloom’s taxonomy. According to this taxonomy, critical thinking includes remembering, understanding, applying, analyzing, evaluating, and creating. Shubina and Kulakli (2020) asserted that critical thinking is the most common way of assessing thoughts, evaluating contentions, managing issues, making decisions, collecting and appraising different data, and concluding about particular principles to give the best solution. Tong et al. (2020) mentioned that critical thinkers reflect, relate, and appraise all features of circumstances or problematic issues. They maintained that this level of thinking incorporates abilities like concentrating on components of a problem or an adverse situation, gathering and coordinating data about the problem, and recalling the understood information. Itmeizeh and Hassan (2020) stated some of the features of critical thinkers, including the followings:

“Purposeful, self-regulatory, self-rectifying, habitually inquisitive, well-informed, trustful of reason, open-minded, flexible, fair-minded in evaluation, honest in facing personal biases, prudent in making judgments, willing to reconsider, clear about issues, orderly in complex matters, diligent in seeking relevant information, reasonable in the selection of criteria, focused in inquiry, and persistent in seeking precise results” (p. 2).

Etemadfar et al. (2020) emphasized that good critical thinking is not an innate or natural ability for most L2 students, but it can be taught through effective pedagogical methods. Some studies have been done on the positive and significant relationship between critical thinking skills and some cognitive skills, such as writing (e.g., Putri, 2018; Esmaeil Nejad et al., 2022), reading comprehension (e.g., Din, 2020; Okasha, 2021), speaking (e.g., Setyarini et al., 2018; Iman and Angraini, 2019), and listening skill, e.g., (Ivanovska and Petkovska, 2019; Erkek and Batur, 2020). In addition, it is important to see the role of individuals’ way of thinking in fostering translation quality. Translation, as a cognitive-emotional skill, requires incorporating different skills and abilities (Hubscher-Davidson, 2013). Kashirina (2014) believed that critical thinking skill is significant in the translation process. She mentioned that teaching critical thinking must be a necessary part of translator professional training because it leads students to acquisition of mature creative thinking, which is crucial for translation problem-solving. She asserted that teaching critical and creative thinking is not an end in itself but a means to improve the quality of the text analysis and, consequently, translation quality, or adequacy. Her study accentuated the role of critical thinking in raising translator’s awareness, and increasing translation quality. Her study implicated the need for students to be facilitated to acquire critical and creative thinking skills in the process of professional training that should incorporate critical and creative thinking training.

### The role of critical thinking in translation performance

Criticality in translation is referred to translations with uncertain, non-institutionalized utilization of the language (Wilss, 1988) or translations in which the choice of a translation alteration is not rule-governed (Kussmaul, 1995). Some investigations have been done on the relationship between critical thinking and translation performance. According to Neubert (1997), criticality is essential to remove the intervention, linguistic or textual, initiated by the source language or the source text. He also argued that “in order to achieve a satisfactory target text, the established rules of correspondence between L1 and L2 need to be criticality extended” (p. 20). Based on the study of Kussmaul (1995), high-quality translations are those in which a translator appropriately employs criticality, and few high-quality translation procedures are represented by the lack of adaptability and reveal the utilization of old techniques in tasks that necessitate a fresh orientation. Using Watson-Glaser Critical Thinking Appraisal (1980), Parham and Fahim (2013) examined the translation trainees’ critical thinking. They scrutinized the role of critical thinking in translation quality. They found that critical thinking significantly predicted translation quality. Their study implicated that teachers should attempt to establish an atmosphere where critical thinking is exercised, and students should pick up relevant skills systematically in a fashion that encourages the application of critical thinking outside the classroom, for all real-life activities. In line with Parham and Fahim (2013), Azin and Heidari Tabrizi (2016) found the relationship between critical thinking and translation performance. They employed California Critical Thinking Skill Test -Form B (CCTST) to assess student translators’ critical thinking skills. They found out that translators with higher levels of critical skills tend to have higher-quality translation performance. They argued that critical thinkers not only outperform in inspecting the message of source language text but also synthesize it more efficiently. Jahromi and Suzani (2016) used Ricketts Critical Thinking Ability questionnaire (2003) to elicit learners’ critical thinking skill. In order to assess learners’ translation quality, they also used Vinay and Darbelnet’s model, which presented two strategies of translation: direct and oblique. The calque, literal and borrowing translation are the constituents of direct strategy. On the other hand, adaptation, transposition, modulation, and equivalence translation are covered in oblique strategies. Their study revealed a significant positive relationship between critical thinking skills and direct strategy of translation of literary texts. Saud (2020), in his study, indicated that student translators’ deductive and inductive reasoning skills, as indicators of critical thinking, significantly predicted their translation performance. He mentioned that translators with higher deductive and inductive cognitive skills outperform in translation performance. He also asserted that translators are required to examine various translations, make a judgment, and translate or select the best translation through inductive reasoning. Moreover, translators are required to employ critical thinking skills to ponder and interpret different translations, and come to a rational conclusion through deductive reasoning. Ghaemi and Sadoughvanini (2020) indicated that translation trainees’ higher-order thinking skills are significantly correlated with their translation quality. They justified their results based on Bloom’s taxonomy. They mentioned that all the constituents of higher-order thinking skills, such as analyzing and creating, have key roles in increasing translation ability and performance. Their study also revealed that the number of translation errors are significantly correlated with analyzing and creating. They argued that the ‘analysis’ component in Bloom’s taxonomy, including breaking information down into parts and different forms and comparing a source text and background knowledge, develops the extra-linguistic knowledge, “i.e. translation errors of translators to a great extent” (p. 21). However, they mentioned that ‘creating’, as another component of Bloom’s taxonomy, connects the new evidence with earlier knowledge or with multiple texts to support a new notion, and create a new reasoning method. They mentioned translation errors have some psycho-physiological constituents, including intellectual inquisitiveness, critical thinking, and cognitive components, highly correlated with translators ‘creating and analyzing skills.

In the field of machine translation, Li (2022) examined the effect of Chinese student translators’ critical thinking on the quality of post-editing in machine translation by estimating the error frequency for each translator. Using Watson-Glaser Critical Thinking Test and Critical Thinking Assessment Test (CAT), he found that learners, with higher levels of critical thinking, have less frequent post-editing errors. His study showed that lower-level critical thinkers tend to have frequent grammatical and, particularly, stylistic errors. They argued that the level of translators’ critical thinking influences the conformity of the meaning of the translated text to the original version, which is a substantial feature of the literary, informational, economic, legal, and technical translation quality. Therefore, it is required to keep the writer’s style and aesthetics of the work. He also argued that other components, such as professional experience and knowledge, can also affect translators’ linguistic and syntactic errors during post-editing authentic texts. He mentioned that translator’s critical thinking can significantly influence the stylistic errors and the ability to select the most standard version of machine translation. He described stylistics as “a sense of language, which is interconnected with critical thinking” (p. 25). Daems et al. (2017) also found that critical thinking is significantly correlated with translation quality, but they asserted that translator’s experience can mediate this correlation. These above-mentioned studies showed the importance of critical thinking on translators’ translation performance.

However, there is a reciprocal relationship between translation students’ critical thinking and translation tasks. Liu (2019) used Bloom’s cognitive hierarchy theory in order to explicate increasing the influence of translation tasks on translation students’ critical thinking. He believed that the integration of memorization, grasping, manipulation, analyzing, evaluation, and creation should be applied in translation courses which can improve translators’ critical thinking skills.

## Implications and suggestions for further research

This conceptual review probed the role of translators’ critical thinking and emotion regulation in their translation performance. Earlier studies have shown a significant relationship between critical thinking and translation quality (e.g., Liu, 2019; Ghaemi and Sadoughvanini, 2020; Saud, 2020; Li, 2022). In other words, translators’ critical thinking facilitates the analysis of the source text and expedites the efficient translation of the message (Azin and Heidari Tabrizi, 2016). Moreover, translations with higher levels of critical thinking are more likely to use deductive and inductive reasoning in the translation process (Saud, 2020). In addition, studies have shown that the sub-components of Bloom’s higher-order thinking skills, including analyzing and creating, are significantly correlated with critical thinking, which fosters translation quality (Liu, 2019; Li, 2022). The results of earlier studies have shown that translators’ use of emotion regulation strategies leads to the efficiency in translation. The studies showed that some emotion regulators, like personality traits and professional expertise, can regulate translators’ emotions, leading to higher translation quality. Translators with higher levels of emotional intelligence, resilience, and intuition, as personality traits, outperform in translation performance.

This conceptual review has some implications for translator trainers, translator trainees, and curriculum designers. Translation trainees can also improve their critical thinking skills by applying some practical ways. Translation trainees can think critically to solve problems so that they meet their objectives. Every decision they make has an objective or purpose attached to it, and identifying exactly what that is, and what they actually want out of it, gives them a starting point to work with. They should ask questions from themselves about the expectation they want to get out of doing tasks. They should also consider the consequences of their decision in tasks. They need to weigh up the possible consequences that may arise from each of their options and go for the one that benefits them most while limiting the negative effects. A good way to do this is by writing a list of pros and cons. By asking themselves to think of every possible positive outcome alongside every possible negative outcome, they can make a much more informed decision. They should spend time on doing research and focusing on learning, and they should adapt themselves to new situations to overcome new situations and improve their critical thinking. There are various techniques and exercises to use critical thinking in classrooms. Caroselli (2009) lists 50 activities. She explained them in three categories: Quick Thinking, Creative Thinking, and Analytical Thinking. Examples of Quick Thinking are brainstorming and perceptual shift. Creative thinking exercises were designed for those competent learners who lack self-confidence and think they should not be expected to come up with critical thinking answers. Analytical thinking, based on the scientific approach of defining a problem, enables learners to overcome a problem. The course should be consistent and logical and students should be aware of what they do. Moreover, theory should be always connected with practice, and there should be constant feedback in the course. In addition, it should have engaging activities in order to trigger critical thinking skills among learners. Translation trainees should engage in doing activities that promote critical thinking skills and require them to reflect, collaborate, ask questions from peers, etc. They should ask meaningful questions since asking questions enhances their critical thinking in learning. Moreover, translators’ critical thinking skills can be improved through social involvement. If they get opportunities to participate in discussions, they must go ahead with it. This will help them encounter different views, examine incoming information, and improve communication skills. They should also practice active learning through understanding and not just by reciting it. Active learning, as a component of the experiential approach, can be attained *via* cooperative learning, visual learning, presentations, etc. The student translators’ critical thinking skills can develop through real-life examples, tales, analogies, and factual stories. Translation students can also foster their emotional intelligence and resilience. They should consider the role of affective factors on their translation performance. It can be mentioned that learners should be assisted to control, adjust, and regulate their emotions to improve their translation quality.

This review can notify translator trainers of the prominence of enhancing translation students’ critical thinking and emotion regulation strategies. Those who teach translation particularly can employ their creativity and include appropriate tasks and classroom activities to enhance students’ critical thinking skills along with their translation ability. Translator trainers should consider students’ attitudes, interests, abilities, and skills in these kinds of tasks and classroom activities and should try to encourage the students to use their critical thinking, and this can be the first step toward enhancing of students’ critical thinking. In order to increase students’ critical thinking, translator trainers can guide the trainees through exercises, provide more opportunities for them to ask different questions, and help them study materials increasing higher-order thinking skills. To keep the students’ attention going, translator trainers can add quizzes, puzzles, and create an appealing experience for students. They should devote time to translating students to participating in active, collaborative learning activities that help them appraise, scrutinize, and synthesize materials. Moreover, they can use classroom debates and appoint learners to involve in debated issue, and help them defend their views. Translator trainers’ effective use of questions and involving students in class discussions over challenging and appealing topics could engage them in critical thinking processes. Therefore, asking appropriate higher-level questions can promote the translation students’ critical thinking. They can also use jigsaw activities by dividing the class into groups. They can have each individual in the group explore a different feature of a broader topic. During class time, trainers can ask trainees to share their findings. They are recommended to employ seminars about translation, and they can get students to participate in class discussions. Critical thinking can be explicitly taught in translation classrooms, as an accelerator of the thinking ability as well as the translation ability of the learners. The explicit instruction of critical thinking, according to the review, can develop higher order thinking in the translation process. It is the translator trainers’ responsibility to encourage learners to use their thinking ability and learn to express themselves critically and creatively. It is believed that translator trainers need to be more flexible in their teaching and try to pay more attention to translation students’ attitudes, interests, and abilities. Moreover, translator trainers should use appropriate tasks, and activities in reading courses to promote critical thinking skills, which then can result in the improvement in reading source texts.

This review has shown that personality traits are regarded as the components which regulate the translation students’ emotions. Translator trainers should be aware of students’ characters, and they should recognize translation students’ needs and help them to find their solutions for translation problems. Emotional intelligence, as a component of personality traits is considered as a regulator of translators’ emotions. The translator trainers are required to provide inspiring and enjoyable translation tasks which provoke their emotional intelligence and reduce language anxiety in their minds. Thus, the provision of enjoyable tasks can regulate translation students’ emotional intelligence to advance their translation performance. These issues dwindle students’ cognitive load and foster their translation quality. Translator trainers can also improve students’ resilience as a regulator of emotion. The role of resilience could be that of influencing the appraisal of threat and thereby the level of anxiety experienced. Higher levels of resilience-related resources might enable individuals to manage any anxiety they experience, thus limiting any adverse effects on performance. Translator trainers can foster translation students’ resilience through positive rapport, instruction of social–emotional skills, developing positive emotional constructs, and building a sense of meaning and purpose.

Moreover, this conceptual review can enlighten those who are involved in planning curriculum for translation students and can equip them with additional information regarding the complex concepts of critical thinking and emotional regulation. Those who develop the curriculum for translation students can include purposeful courses in translation. The specific focus of these courses should be on critical thinking skills and emotions, the purpose of such courses should be the training of translators with high critical thinking and emotion regulation strategies. In other words, the focus of curriculum designers should be on critical questioning, critical reading, critical and creative writing, and critical listening in all curriculum areas.

Future research should consider the potential effects on the quality of translation. Future studies could investigate the association between critical thinking and different types of translation texts. Moreover, it is necessary to investigate how translation quality is influenced by translation students’ positive emotional constructs, such as foreign language enjoyment, engagement, grit, self-efficacy, pedagogical love, and well-being. Further studies should investigate the influence of factors like academic capabilities, learner style, and negative emotional states, such as foreign language anxiety, apprehension, boredom, and burnout, on translation quality. Moreover, the relationship between translators’ critical thinking and their emotion regulators, such as resilience, emotional intelligence, intuition, and professional expertise should be investigated in the future. Finally, the effect of some variables, such as age, gender, education, intercultural communication experiences, and economic status, on student translators’ critical thinking skill and their strategy in regulating emotions can also be considered in the future.

## Author contributions

The author confirms being the sole contributor of this work and has approved it for publication.

## Funding

This study received funding from Academic Degrees & Graduate Education Reform Project of Henan Province, “Research and Practice of the Construction of Teaching Quality Assurance System for MTI with Aviation Characteristics” (Project number: 2021SJGLX246Y) and Postgraduate Education Reform and Quality Improvement Project of Henan Province, “Project of Joint Training Base for Graduate Students in Henan Province” (Project number: YJS2022JD44).

## Conflict of interest

The author declares that the research was conducted in the absence of any commercial or financial relationships that could be construed as a potential conflict of interest.

## Publisher’s note

All claims expressed in this article are solely those of the authors and do not necessarily represent those of their affiliated organizations, or those of the publisher, the editors and the reviewers. Any product that may be evaluated in this article, or claim that may be made by its manufacturer, is not guaranteed or endorsed by the publisher.
